# Cerebrovascular intervention therapy worked positively in one patient with severe cerebral venous sinus thrombosis due to hyperthyroidism: a case report and review of the literature

**DOI:** 10.1186/s13256-022-03463-y

**Published:** 2022-06-26

**Authors:** Jia Jia, Gangmin Xi, Wei Fan, Guiping Wang, Junwen Li, Jing Huang

**Affiliations:** 1 Department of Neurology, Shanghai Xuhui District Central Hospital, 966 Middle Huaihai Road, Xuhui District, Shanghai, China; 2 Department of Neurology, Zhongshan Hospital, Fudan University, Shanghai, China

**Keywords:** Cerebrovascular disease, Cerebral venous sinus thrombosis, Hyperthyroidism, Cerebrovascular intervention, Case report

## Abstract

**Background:**

With further understanding of cerebral venous sinus thrombosis, hyperthyroidism has gradually been revealed as a rare predisposing factor for cerebral venous sinus thrombosis, which may present as more compact clots and resistance to fibrinolysis, also known as a predictor of worse outcomes. For patients with severe cerebral venous sinus thrombosis, proper treatment method should be initiated as soon as possible since they may deteriorate rapidly.

**Case presentation:**

In this case report, we present a 32-year-old Mongoloid woman admitted with progressive headache, impaired consciousness, and right limb weakness, diagnosed with cerebral venous sinus thrombosis caused by hyperthyroidism. A cerebrovascular intervention with local thrombolytic infusion was performed at the site of thrombosis, followed by dilatation with balloon and thrombus aspiration in venous sinus, with partial recanalization observed and anticoagulation given as a next step. After cerebrovascular intervention, the patient’s condition improved rapidly and she was discharged with her National Institute of Health Stroke Scale score being decreased from 17 to 2.

**Conclusions:**

When patients with hyperthyroidism suffer from headache, progressive disturbance of consciousness, seizures, and other symptoms, the presence of cerebral venous sinus thrombosis should be considered and corresponding examinations should be performed as soon as possible. For patients with severe cerebral venous sinus thrombosis, cerebrovascular intervention might be a safe and effective approach if conventional management fails.

## Background

Cerebral venous sinus thrombosis (CVST), accounting for 0.5–1.0% of strokes, is the occlusion of cerebral veins that causes venous infarction and intracerebral hemorrhage. Widely accepted risk factors of CVST include coagulation disorders (genetic or acquired), inflammatory reaction, autoimmune diseases, use of oral contraceptive, hormonal changes, puerperium or pregnancy states, and systemic diseases. As recommended by the American Heart Association/American Stroke Association (AHA/ASA) and European Federation of Neurological Societies (EFNS) guidelines, anticoagulation should be administrated immediately once diagnosed as CVST, even in the presence of minor hemorrhage [1, 2]. However, about 10% of patients had poor outcome despite anticoagulation therapy. In this paper, we report a case of severe CVST in whom conventional anticoagulants did not work and specific interventional therapeutic module achieved a great outcome.

## Case presentation

**Fig. 1:**
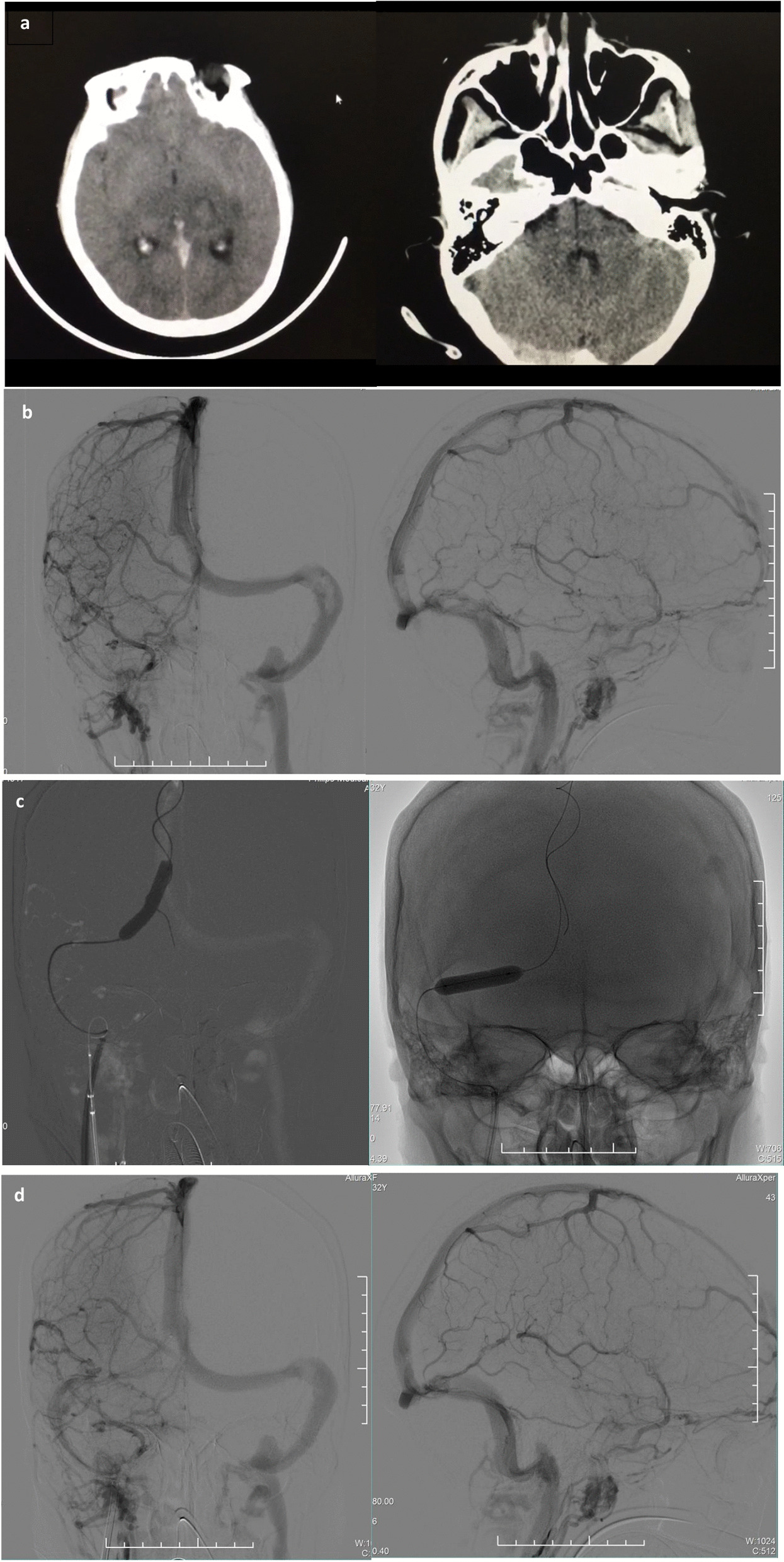
**a** Noncontrast brain CT showing increased density of straight sinus and right transverse sinus. **b** The anterior and lateral positions of the right internal jugular vein stage: nonvisualization of right transverse, sigmoid sinus, and straight sinus, and identification of large thrombus among the junction of the superior sagittal sinus, the left transverse sinus, and the sigmoid sinus, with severe stenosis of the corresponding vein lumens.** c** Balloon-dilated sinus confluence and transverse sinus. **d** Final angiography showing improvement of the right transverse sinus and the sigmoid sinus compared with the front, and nonvisualization of the straight sinus

A 32-year-old Mongoloid female with headache, progressive disturbance of consciousness, and right limb weakness was transferred to our hospital. On arrival, she was in a mild coma with dilated pupils. Glasgow Coma Scale (GCS) score was 9 (4 + 4 + 1). No movement but muscle retraction was observed in the right limb and the Babinski sign is positive. The National Institute of Health Stroke Scale (NIHSS) score was 17. The patient was a fruit seller without previous chronic diseases, and denied smoking and drinking history, toxic and drug exposure, and family history. Noncontrast brain computed tomography (CT) and CT venography scan in another hospital showed CVST in straight sinus and sagittal sinus (Fig. 1a). Subcutaneous injection of weight-based low-molecular-weight heparin was immediately administered, yet symptoms deteriorated within 2 days.

After admission, cerebrovascular intervention was performed immediately, and the images indicated nonvisualization of right transverse, sigmoid sinus, and straight sinus, and large thrombus was identified among the junction of the superior sagittal sinus, the left transverse sinus, and the sigmoid sinus, with severe stenosis of the corresponding vein lumens (Fig. 1b). Urokinase was injected into right transverse sinus and upper sagittal sinus via microcatheter. After the operation, the microcatheter and the sheath were kept for alternate use of urokinase and alteplase in the next 2 days. Unfortunately, the patient’s symptoms did not improve significantly. We performed a second cerebral angiography and found nonvisualization of the straight sinus and the right transverse, while the large thrombus almost disappeared. Using balloon dilation and thrombus aspiration, we found that the visualization of right transverse sinus and sigmoid sinus was improved, while that of the straight sinus was not (Fig. 1c, d). After intervention, full-dose anticoagulation therapy was administrated, despite large bruises on both upper limbs and slight hemorrhage in the thalamus. The patient’s consciousness state was gradually improved within 2 days. Her later laboratory examination showed that serum thyroid-stimulating hormone was low with high free triiodothyronine (T3), free thyroxine (T4), and antibodies, but she was never diagnosed or treated. The department of endocrinology was consulted, she was diagnosed with hyperthyroidism (Graves’ disease), and corresponding drug treatment was carried out. She was discharged with her NIHSS score decreased to 2, diagnosed with hyperthyroidism and refractory CVST. Three months later, she complained of occasional numbness in right limb with NIHSS score of 0. Magnetic resonance venography (MRV) showed that sagittal sinus, transverse sinus, and sigmoid sinus visualized well, although the straight sinus was less clear.

## Discussion and conclusions

Before 2005, the relationship between thyrotoxicosis and CVST was not clear, with fewer than 20 cases reported. Some authors supposed that variations of hemodynamic factors, dehydration, and stasis of venous blood flow caused by thyrotoxicosis might contribute to genesis of CVST [3].With further understanding, hyperthyroidism has gradually been revealed as a rare predisposing factor for CVST. Hooper proved that endogenous hyperthyroidism is associated with more compact clots and resistance to fibrinolysis *in vitro*, which may be one of the reasons why CVST due to hyperthyroidism often has a poor prognosis [4]. When patients with hyperthyroidism suffer from headache, progressive disturbance of consciousness, seizures, and other symptoms, the presence of CVST should be considered, and MRV or other corresponding examinations should be performed as soon as possible.

Our patient’s consciousness was impaired owing to increased intracranial pressure caused by CVST, and previous anticoagulant treatments showed no response. An emergency cerebrovascular intervention seemed the only effective and reasonable approach at that time and was proven to be quite effective in this patient. A cohort study suggests that cerebrovascular intervention might be a safe and effective approach for patients with CVST with cerebral edema when conventional management fails [5]. Intrasinus thrombolysis (IST), direct mechanical thrombectomy (MT) with or without thrombolysis, suction, and venous sinus stent implantation are now more popularly used than previously as salvage treatments of severe CVST [2, 6]. These conclusions are consistent with the guidelines of the AHA, which suggest that interventional techniques may be reasonable when clinical condition deteriorates because of venous infarction or corresponding ICH caused by anticoagulation [2]. However, the more we learned about the intervention therapy for patients with severe CVST, the more problems appeared. For example, the choice of thrombolysis medicine, selection of appropriate dose, the proper time window of interventional therapy, and the operation method are all debated.

Our case provided a new combination of operations and showed effectiveness, suggesting that the choice of exact operation method can be adjusted in a timely manner according to the specific situation and the patient’s response to treatment. In the future, large, well-designed prospective randomized trials are needed to provide more information on the safety and validity of interventional therapy. At the same time, better tools for early identification of patients with high-risk CVST unlikely to respond well to standard anticoagulation therapy are urgently required.

## Data Availability

The data are available via contacting the corresponding author.
